# 4184 Implementation of an Opt-Out recruitment policy at Duke University School of Medicine

**DOI:** 10.1017/cts.2020.272

**Published:** 2020-07-29

**Authors:** Michelle Mack, Jamie Roberts, Dalia Mack, Steven Shipes, Stephanie A. Freel, Marissa Stroo, Roy McDonald, Denise Snyder

**Affiliations:** 1 Duke University; 2 Duke Office of Clinical Research; 3 Duke University School of Medicine

## Abstract

OBJECTIVES/GOALS: In March 2019, Duke updated recruitment guidelines and adopted an “Engagement” policy that requires patients to ‘opt-out’ of communications regarding potential research participation. This created an opportunity to evaluate recruitment for ongoing clinical studies pre and post implementation. METHODS/STUDY POPULATION: Implementation of the new policy required new training for study teams, modification to recruitment plans, and expansion of ongoing efforts to improve direct-to-patient messaging through EPIC/MyChart tools. The impact of this new policy on overall recruitment was monitored and characterized both prior to and after implementation of the policy. Customized MyChart messages have been generated for over 22 studies, with a total of 41,386 messages sent to potential participants. RESULTS/ANTICIPATED RESULTS: Only a small number of study teams have modified their recruitment plans with transition to the new policy. This may be related to lack of understanding about policy implementation, potential recruitment opportunities, required training, resource limitations, etc. However, our case study, TMIST, had an 48% improvement in average enrollment within the first 2 months of implementation, and an almost 40% improvement in recruitment efficiency. Since becoming an “opt-out” institution, 11 study teams have implemented direct-to-patient recruitment via the MyChart patient portal. One unintended consequence we’ve noted is several different study invitations to potential participants within some patient populations. DISCUSSION/SIGNIFICANCE OF IMPACT: The new policy allows study teams to engage in direct-to-patient outreach, leading to an increase in enrollment for some studies. Incorporation of direct-to-patient messaging strategies can be a cost and resource saving measure to improve recruitment. The need to recruit similar populations demonstrated that strategic, thoughtful approaches are needed.

